# Improving prediction of heart transplantation outcome using deep learning techniques

**DOI:** 10.1038/s41598-018-21417-7

**Published:** 2018-02-26

**Authors:** Dennis Medved, Mattias Ohlsson, Peter Höglund, Bodil Andersson, Pierre Nugues, Johan Nilsson

**Affiliations:** 1 Department of Computer Science, Lund University, Lund, Sweden; 2 Department of Astronomy and Theoretical Physics, Computational Biology and Biological Physics, Lund University, Lund, Sweden; 3 Department of Laboratory Medicine Lund, Clinical Chemistry and Pharmacology, Lund University, Lund, Sweden; 4 Department of Clinical Sciences Lund, Surgery, Lund University and Skåne University Hospital, Lund, Sweden; 5 Department of Clinical Sciences Lund, Cardiothoracic Surgery, Lund University and Skåne University Hospital, Lund, Sweden

## Abstract

The primary objective of this study is to compare the accuracy of two risk models, International Heart Transplantation Survival Algorithm (IHTSA), developed using deep learning technique, and Index for Mortality Prediction After Cardiac Transplantation (IMPACT), to predict survival after heart transplantation. Data from adult heart transplanted patients between January 1997 to December 2011 were collected from the UNOS registry. The study included 27,860 heart transplantations, corresponding to 27,705 patients. The study cohorts were divided into patients transplanted before 2009 (derivation cohort) and from 2009 (test cohort). The receiver operating characteristic (ROC) values, for the validation cohort, computed for one-year mortality, were 0.654 (95% CI: 0.629–0.679) for IHTSA and 0.608 (0.583–0.634) for the IMPACT model. The discrimination reached a C-index for long-term survival of 0.627 (0.608–0.646) for IHTSA, compared with 0.584 (0.564–0.605) for the IMPACT model. These figures correspond to an error reduction of 12% for ROC and 10% for C-index by using deep learning technique. The predicted one-year mortality rates for were 12% and 22% for IHTSA and IMPACT, respectively, versus an actual mortality rate of 10%. The IHTSA model showed superior discriminatory power to predict one-year mortality and survival over time after heart transplantation compared to the IMPACT model.

## Introduction

Heart transplantation (HT) is a life-saving operation for patients with end-stage heart disease. Despite this reality, the transplantation number does not increase over the years. One of the most limiting factors is the lack of donor organs and a conservative allocation policy that results in the loss of about half of the organs being offered^1^. An improved prediction of the outcome would augment the confidence in the post-transplantation performance and make it possible to optimise the allocation of organs. Furthermore, it would enable practitioners to determine the risk of early and late graft dysfunction more accurately and improve donor and recipient management.

Although there exist several survival models within cardiac surgery, currently there is no accepted tool for estimating the outcome after heart transplantation. In recent years, some risk score algorithms designed to predict post-transplantation performance have been developed, which almost all have been derivate on the single national, multi institutional United Network for Organ Sharing (UNOS) registry^2^. The most notable ones are: Donor Risk Index (DRI), Risk Stratification Score (RSS), and Index for Mortality Prediction After Cardiac Transplantation (IMPACT)^3–5^. The IMPACT model has additionally been validated on the International Society of Heart and Lung Transplantation (ISHLT) registry and showed an acceptable accuracy in predicting mortality. Recently a multinational model, the International Heart Transplantation Survival Algorithm (IHTSA), developed on the ISHLT registry was published^6^. This model was designed to predict both short-term and long-term mortality and, in contrast to previous models, it utilises deep learning techniques. The results it obtained showed an improved discrimination compared with the DRI, RSS, and IMPACT models. However, the validation was performed on the ISHLT registry, which was also used for the development of the model^6^.

Even if the validation cohort was separated from the derivation cohort, the IHTSA model might be biased towards this registry.

The aim of this study was to determine the most suitable risk stratification model for heart transplantation by applying the IMPACT and IHTSA algorithms to the UNOS registry.

## Results

### Characteristics of the Study Population

The preoperative characteristics of the recipients are listed in Table 1 and for the donors in Table 2. The number of adult HT with a follow-up time of at least one year, from January 1997 to December 2011, was of 27,860, corresponding to 27,705 patients. Over the time span, the cumulative sum of follow-up years was of 165,206. The median survival time was 12 years (Interquartile Range [IQR]: 5–16). The one-year mortality was of 13% (n = 3,561). The average age of the recipients was 52 ± 13 years, with a range from 18 to 78 years. Most of the recipients were males 76% (n = 21,151). Multi-organ transplants were marginal (2.5%). The number of transplants contained in the derivation cohort was of 22,263, and the number of transplants in the test cohort was of 5,597.

**Table 1:** The recipient features used in the IMPACT and IHTSA Models.

Feature	N	Time era 1997–2008	Time era 2009–2011	p-Value	IMPACT	IHTSA
		(n = 22,263)	(n = 5,597)			
**Demographic data**						
Age (years)	27,860	52 ± 13	53 ± 13	0.001		✓
Age >60 years	27,860	5,707 (26%)	1,809 (32%)	0.001	✓	
Female gender	27,860	5,298 (24%)	1,411 (25%)	0.029	✓	✓
Height (cm)	27,740	174 ± 10	174 ± 10	0.835		✓
Weight (kg)	27,760	80 ± 17	82 ± 17	0.001		✓
Race: African American	27,860	3,324 (15%)	1,103 (20%)	0.001	✓	
Diagnosis						
Ischemic cardiomyopathy	27,859	9,976 (45%)	2,793 (50%)	0.001	✓	✓
Non-ischemic cardiomyopathy	27,859	10,247 (46%)	2,119 (38%)	0.001		✓
Congenital	27,859	518 (2%)	149 (3%)	0.159	✓	✓
Other	27,859	852 (3%)	247 (4%)	0.001	✓	
Graft failure	27,859	669 (3%)	197 (4%)	0.058		✓
Diabetes mellitus^#^	27,597	4,735 (22%)	1,500 (27%)	0.001		✓
Hypertension^†^	17,876	7,108 (40%)	—			✓
Infection within two weeks^‡^	26,543	2,333 (11%)	594 (11%)	0.550	✓	✓
Antiarrhythmic drugs prior transplant	17,266	6,371 (37%)	—			✓
Amiodarone prior to transplant	17,530	4,726 (27%)	—			✓
Dialysis prior to transplant	27,002	706 (3%)	185 (3%)	0.510	✓	
Previous blood transfusion	15,221	5,285 (35%)	27 (29%)	0.247		✓
Previously transplanted*	27,860	680 (3%)	199 (4%)	0.067		✓
Previous cardiac surgery	14,069	1,866 (22%)	1,483 (27%)	0.001		✓
ICU	27,860	7,991 (36%)	1,493 (27%)	0.001		✓
Mechanical ventilation	27,860	625 (3%)	166 (3%)	0.532	✓	✓
ECMO	27,860	90 (0.04%)	48 (1%)	0.001		✓
IABP	27,860	1193 (5%)	263 (5%)	0.039	✓	✓
Ventricular assist device	24,357	4,665 (25%)	2,191 (39%)	0.001		✓
Early generation^a^	6,856	911 (20%)	114 (5%)	0.001	✓	
Late generation^b^	6,856	536 (11%)	1,610 (74%)	0.001	✓	
Other/Unknown	6,856	3,218 (69%)	467 (21%)	0.001		
Temporary circulatory support^c^	27,860	209 (1%)	113 (2%)	0.001	✓	
Transplant era						
1996–2000	27,860	7781 (35%)	—			✓
2001–2005	27,860	8981 (40%)	—			✓
>2005	27,860	5501 (25%)	5,598 (100%)	0.001		✓
**Hemodynamic status**						
PVR (wood units)	21,782	2.5 ± 1.8	2.4 ± 1.8	0.205		✓
SPP (mmHg)	25,100	43 ± 14	42 ± 14	0.001		✓
**Laboratory values**						
Creatinine (mg/dl)	27,027 1	1.4 ± 0.8	1.3 ± 0.8	0.038		✓
Creatinine clearance (mL/min)						
30–49	27,054	2,964 (14%)	698 (12%)	0.008	✓	
<30	27,054	674 (3%)	189 (3%)	0.376	✓	
Serum bilirubin (mg/dl)	26,224	1.3 ± 2	1.2 ± 2	0.001		✓
1.00–1.99	26,224	6,117 (30%)	1,562 (28%)	0.102	✓	
2.00–3.99	26,224	1261 (6%)	300 (5%)	0.070	✓	
≥4	26,224	1314 (6%)	297 (5%)	0.007	✓	
**Immunology status**						
PRA > 10%	18,351	1,113 (8%)	1,114 (20%)	0.001		✓
HLA-DR, 2 mismatch	23,858	10,289 (55%)	2,746 (55%)	0.906		✓
**Recipient blood group**						
A	27,860	9,543 (43%)	2,313 (41%)	0.036		✓
B	27,860	3,040 (14%)	795 (14%)	0.343		✓
AB	27,860	1,143 (5%)	295 (5%)	0.597		✓
O	27,860	8,549 (38%)	2,198 (39%)	0.092		✓

N, number of transplants with non-missing values. n, total number of transplants. Qualitative data are expressed as n (%), and quantitative data as mean ± SD. ^#^Drug or insulin treated diabetes mellitus.

^†^Drug treated systemic hypertension. ^‡^Infection requiring intravenous antibiotic therapy within two weeks prior to transplant. *Previous transplant—previous kidney, liver, pancreas, pancreas islet cells, heart, lung, intestine and/or bone marrow transplant. ^a^Early generation includes para and intracorporeal pulsatile VADs: Abiomed AB5000, Heartmate I, XE, and XVE, ThortecIVAD, Toyobo, Medos and LionHeart. ^b^Later generation continuous VADs including Heartmate II, Jarvik, Micromed, Debakey, and VentrAssist. ^c^Includes ECMO and [or] extracorporeal VADs: Abiomed BVS5000, Bio-Medicus, TandemHeart, and Levitronix/Centrimag. ECMO, extracorporeal membrane oxygenation; ICU, intensive care unit; IHTSA, international heart transplantation survival algorithm; IMPACT, index for mortality prediction after cardiac transplantation; HLA, human leukocyte antigen; PRA, panel reactive antibody; PVR, pulmonary vascular resistance; SD, standard deviation; SPP, systolic pulmonary pressure. The t-test and chi-squared test was used for continuous respectively categorical values.

**Table 2:** The donor features used in the IHTSA model.

Feature	N	Time era 1997–2008	Time era 2009–2011	p-Value	IMPACT	IHTSA
		(n = 22,263)	(n = 5,597)			
**Demographic data**						
Age (years)	27,075	32 ± 12	32 ± 12	0.515		✓
Female gender	27,860	6,546 (29%)	1,645 (29%)	0.979		✓
Weight (kg)	27,838	79 ± 19	82 ± 19	0.001		✓
Duration of ischemia (min)	26,029	189 ± 63	194 ± 10	0.001		✓
CODD: Head Trauma	27,825	13,733 (62%)	3,068 (55%)	0.001		✓
CODD: Cerebrovascular event	27,825	5,894 (27%)	1,297 (23%)	0.001		✓
**Donor blood group**						
A	27,859	8,232 (37%)	1,983(35%)	0.030		✓
B	27,859	2,284 (10%)	617 (11%)	0.102		✓
AB	27,859	477 (2%)	125 (2%)	0.682		✓
O	27,859	11269 (40%)	2,873 (51%)	0.001		✓
Recipient-donor weight ratio	27,739	1.03 ± 0.22	1.02 ± 0.20	0.001		✓
Recipient-donor height ratio	27,660	0.998 ± 0.06	0.999 ± 0.06	0.068		✓

N, number of transplants with non-missing values. n, total number of transplants. Qualitative data are expressed as n (%), and quantitative data as mean ± SD. CODD, cause of donor death; IHTSA, international heart transplantation survival algorithm; IMPACT, index for mortality prediction after cardiac transplantation. The t-test and chi-squared test was used for continuous respectively categorical values.

### IMPACT versus IHTSA

The IHTSA model includes 32 recipient risk variables, while the IMPACT model has 18 variables; five of these variables are shared between the models: female gender, diagnosis: ischemic cardiomyopathy, diagnosis: congenital, infection within two weeks, and mechanical ventilation. Additionally, IHTSA also has 11 donor variables, while IMPACT has no donor variables.

**Figure 1:**
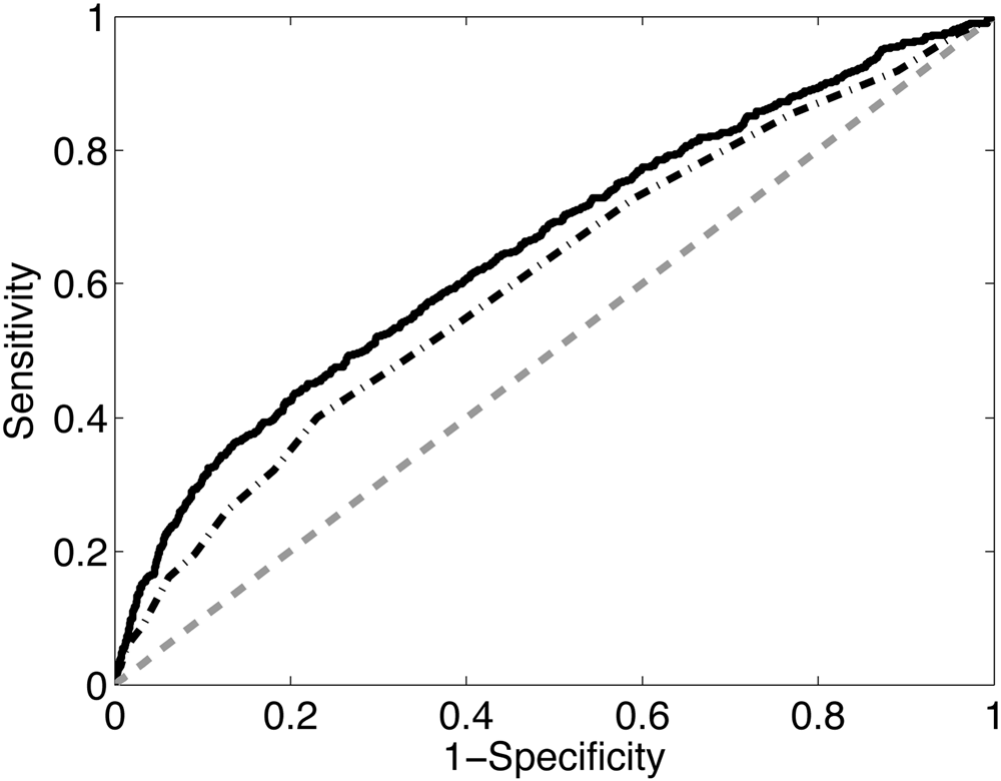
The ROC curves show the sensitivity of prediction of one-year mortality vs. 1-specificity for the IMPACT (short-long dashed line) and the recalibrated IHTSA (solid line) risk algorithms is plotted on the test cohort (2009–2011). The gray dashed line represents the absence of discrimination.

We evaluated the original IHTSA model in the test cohort (2009–2011) for one-year mortality; it had an area under receiver operating characteristic (AUROC) of 0.643 (95% CI: 0.619–0.667), while IMPACT had an AUROC of 0.608 (0.583–0.634), *P* = 0.004, see Table 3. As shown in Fig. 1 and Table 3, the recalibrated IHTSA model has a significantly higher discrimination compared with the IMPACT model for one-year mortality, *P* = 0.001, corresponding to an error reduction of 11.7%. Harrell’s C-index for the recalibrated IHTSA compared with IMPACT was substantially larger, as shown in Table 4, with about a 4% absolute difference for the later time era. This corresponds to an error reduction of 10.3%. On the time era 1997–2008, on which the models were trained using 5-fold cross-validation technique, the recalibrated IHTSA had an AUROC of 0.688 (0.678–0.699), and IMPACT had 0.606 (0.595–0.617) for one-year mortality, *P* = 0.001, Table 3. The absolute difference in C-index was 5% higher for the IHTSA model compared with the IMPACT model, *P* < 0.001, Table 4.

**Table 3:** The AUROC for one-year mortality for the different cohorts using IMPACT and IHTSA respectively.

Time era	AUROC (95% CI)				
	IMPACT	IHTSA	*P*-Value	IHTSA cal.	*P*-Value
1997–2008	0.61 (0.59–0.62)	0.66 (0.64–0.67)	0.001	0.69 (0.68–0.70)	0.001
2009–2011	0.61 (0.58–0.63)	0.64 (0.62–0.67)	0.004	0.65 (0.63–0.68)	0.001

AUROC, area under the receiver-operating curve; CI, confidence interval; IHTSA, international heart transplantation survival algorithm; cal, the recalibrated version; IMPACT, index for mortality prediction after cardiac transplantation.; *P*, probability that the result is the same as IMPACT.

**Table 4:** The Harrells C-index for survival for the different cohorts using IMPACT and IHTSA respectively.

Time era	C-index (95% CI)				
	IMPACT	IHTSA	*P*-Value	IHTSA cal.	*P*-Value
1997–2008	0.56 (0.56–0.56)	0.59 (0.59–0.60)	0.001	0.62 (0.61–0.62)	0.001
2009–2011	0.58 (0.56–0.61)	0.61 (0.59–0.63)	0.002	0.63 (0.61–0.65)	0.001

CI, confidence interval; IHTSA, international heart transplantation survival algorithm; cal, the recalibrated version; IMPACT, index for mortality prediction after cardiac transplantation; *P*, probability that the result is the same as IMPACT.

**Figure 2:**
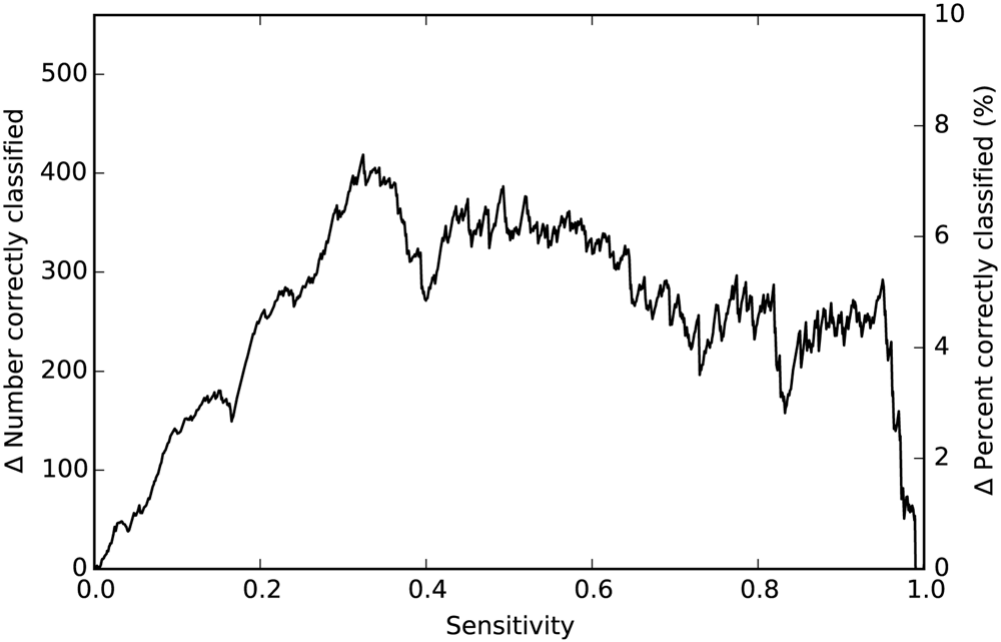
The sensitivity of prediction of one-year mortality versus the total number of additional correctly classified patients by IHTSA compared with IMPACT, both in absolute numbers and percentage, plotted on the test cohort (2009–2011).

We analysed the sensitivity of both models relatively to the deceased patients after one year at the levels of 25%, 50%, and 75%. Out of the transplants in the test cohort (N = 5,597), the numbers of correctly classified patients after one year were 4,812, 3,890, and 2,582 patients respectively for IHTSA, and 4,539, 3,396, and 2,140 patients respectively for IMPACT. See Fig. 2 for a graph of the difference in correctly classified patients.

**Figure 3:**
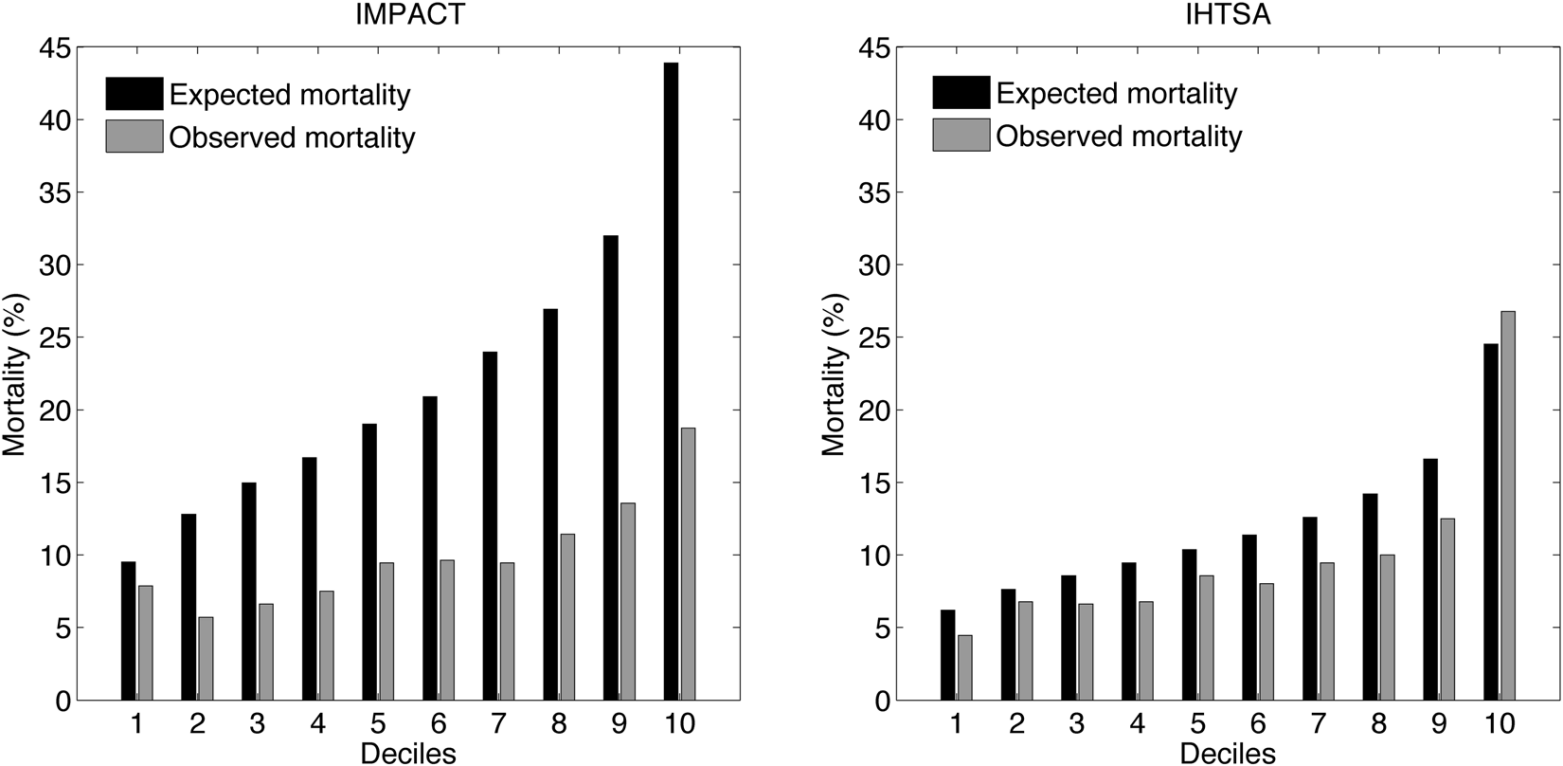
The observed (gray bars) and expected mortality (black bars), in percent, for each decile, for the IMPACT and IHTSA models, in the test cohort (2009–2011). The patients are divided into deciles according to their expected mortality, and the observed mortality was derived for each decile.

We furthermore compared the predicted one-year mortality rate for IMPACT and IHTSA, with the true mortality rate. The predicted one-year mortality for the second time-era (test cohort) was 12% and 22% for the recalibrated IHTSA and IMPACT, respectively, versus an actual mortality rate of 10%. The Hosmer-Lemeshow (HL) chi-square for one-year, using ten groups, was of 40 in the IHTSA model and 101 for the IMPACT model, both with a *P*-value less than 0.05. As shown in the calibration plot, Fig. 3, the predictive mortality compared with actual mortality was more consistent over all deciles for the ITHSA model compared with the IMPACT model.

To evaluate difference in methodology approach (deep learning versus logistic regression), we performed two additional experiments. We quantify the difference between the deep learning technique used by the IHTSA model and the more traditional logistic regression approach used by the IMPACT model, by letting the two systems use identical features. The second experiment was to assess the difference between a model that include and exclude donor variables.

As shown in Tables 5 and 6, a recalibrated IHTSA model including only the same risk variables as the IMPACT model still showed a substantial improvement in the AUROC (about 2%) and C-index in the test cohort compared with the IMPACT model. The recalibrated IHTSA model excluding the donor variables showed a decrease in discrimination compared with the original IHTSA model, however the difference was minor, producing nearly the same AUROC.

**Table 5:** The AUROC for one-year mortality for the test cohort (2009–2011) using an artificial neural network model derived on the derivation cohort (1997–2008) with IMPACT features only (ANN I) and with IHTSA recipient features only (ANN II).

Time era	AUROC (95% CI)				
	IMPACT	ANN I	*P*-Value	ANN II	*P*-Value
2009–2011	0.61 (0.58–0.63)	0.63 (0.60–0.65)	0.027	0.65 (0.63–0.68)	0.001

AUROC, area under the receiver-operating curve; CI, confidence interval; IHTSA, international heart transplantation survival algorithm; IMPACT, index for mortality prediction after cardiac transplantation.; *P*, probability that the result is the same as IMPACT.

**Table 6:** The Harrells C-index for one-year mortality for the test cohort (2009–2011) using an artificial neural network model derived on the derivation cohort (1997–2008) with IMPACT features only (ANN I) and with IHTSA recipient features only (ANN II).

Time era	C-index (95% CI)				
	IMPACT	ANN I	*P*-Value	ANN II	*P*-Value
2009–2011	0.58 (0.56–0.61)	0.60 (0.58–0.62)	0.002	0.62 (0.60–0.64)	0.001

CI, confidence interval; IHTSA, international heart transplantation survival algorithm; IMPACT, index for mortality prediction after cardiac transplantation.; *P*, probability that the result is the same as IMPACT.

## Discussion

The purpose of this study was to compare the IMPACT and IHTSA models with regards to the prediction accuracy of one-year mortality on the UNOS database. There exist some biases in both models when used on the UNOS data set for the time era 1997–2008. Because IMPACT was developed on these data and IHTSA on the ISHLT dataset, which consists in part of the same UNOS data, the models may be subjected to a non-negligible overfit to the data, skewing the result towards a more positive value. Therefore, we chose to validate the models on a later time era, which has no overlapping patients with the training set.

The results show that the IHTSA model exhibited improved performance and accuracy compared to the IMPACT model. Even though IMPACT was designed to predict one-year mortality and IHTSA was created for long-term survival, IHTSA shows better discrimination on one-year mortality.

This study could also prove the benefits of using deep learning modelling techniques. Such techniques are inspired by the human brain. They consist of a network of “neurons” that emulate the properties of their real counterparts. Using multiple processing layers makes it possible to learn representations of data with multiple levels of abstraction^7^. These methods have improved the state-of-the-art in speech recognition, visual object recognition, object detection and many other domains^8^.

Our results show that the IHTSA model can be applied to predict short-term mortality with greater accuracy than a more traditional risk-based model based on logistic regression. Although the comparison of ROC curves to evaluate models in a statistically valid manner is controversial, the ROC curve is currently the most developed statistical tool for describing performance^9,10^. The improvements seen can be explained by the difference in the variable selection, such as the absence of donor risk factors in the IMPACT model, but also by the the neural network’s ability to handle interactions between variables and nonlinearities. An increased donor age has in previous reports been shown to have a negative influence on short-term survival^6,11^. To examine this, we compared the difference of the deep learning model and the logistic regression model using the same variables. Here, we show a substantial improvement when using the deep learning approach compared with the traditional approach. Furthermore, we could show that the predictive availability for the deep learning model was less dependent on the variables included compared with a standard model. Donor variables showed to be of less importance than expected. A possible explanation for that may be the deep learning technology has an increased ability to identify new patterns with the data it has available. It is interesting to note that the two models do not show a considerable overlap of features. Only five features are shared by the two models out of 18 for IMPACT and 43 for IHTSA. If we compare the overlapping variables with the seven most important variables for IHTSA, we find that three of them are shared: age, diagnosis, and mechanical ventilation^6^.

One disadvantage of the deep learning technique is that it yields a black box model with a limited ability to explicitly identify possible causal relationships. Logistic regression, on the contrary, makes it feasible to determine the strongly predictive variables based on the size of the coefficients. To cope with the lack of a well-established method for interpreting the weights of a connection matrix in a neural network, the developers of the IHTSA algorithm used a classification and regression tree (CART), fitted to the predicted median survival time, to assess the relative importance of the features^6^. Furthermore, the web-based calculator (http://ihtsa.cs.lth.se) makes it possible to estimate the survival on a computer or mobile device.

During 2011, approximately 17,000 donors were reported^12^. Unfortunately, not more than one-third of all donors could be utilised for heart transplantation. One explanation for this may be the uncertainty in the risk of early and late graft dysfunction, which means that some suitable donors are not accepted. Although there are many donor predictors of allograft discard in the current era, these characteristics seem to have little effect on recipient outcomes when the hearts are transplanted, which also is confirmed in this study^13^. A more liberal use of cardiac allografts with relative contraindications may be warranted. A calculator would allow us to conveniently perform batch estimation of survival for multiple patients at the same time. This would allow the IHTSA model to be used as a virtual recipient-donor matching tool that models survival for potential recipients on a waiting list when there is a donor heart available. This could potentially increase the number of organs that could be used compared with a traditional criterion-based model^6^. Additionally, it will make it easier for other research groups to validate the model.

The results of this study carry limitations associated with the retrospective analysis of a registry database, the quality of the source data, the number of missing data, and the lack of standardization associated with multicenter studies (such as different immunosuppressive regimens and different matching criteria). However, those limitations are the same for both models. Even if a comparison of risk models remains controversial, the C-index is probably the best statistical tool for describing performance. A C-index of <0.7 may seem low, but it should be kept in mind that the IHTSA model predicts long term survival, and to the best of our knowledge, it is higher than previously reported studies.

## Conclusions

In this study, we have shown that a flexible nonlinear artificial neural network model (IHTSA), utilising deep learning techniques, exhibits better discrimination and accuracy than a more traditional risk score model (IMPACT) for predicting one-year mortality. We made public the results of this model in the form of a web-based batch calculator that could be used as a virtual recipient-donor matching tool. This is a first step in the implementation of a deep learning architecture for transplantation data that, we hope, will pave the way for further improvements and an even more accurate model.

## Materials and Methods

### Data Source

The data set of heart transplant patients was obtained from the UNOS database. UNOS is a non-profit organisation that administers the only Organ Procurement and Transplantation Network (OPTN) in the United States of America^14^. The database contains data from October 1, 1987, onwards and includes almost 500 variables that encompass recipient, donor, and transplant information. It consists of both deceased- and living-recipient transplants. The Ethics Committee for Clinical Research at Lund University, Sweden approved the study protocol. The data was anonymized and de-identified prior to analysis and the institutional review board waived the need for written informed consent from the participants.

### Study Population

We included all the adult HT patients (>17 years) from January 1997 to December 2011. The latest annual follow-up was on September 30, 2013. The data set was divided into two temporal cohorts: transplantation done before 2009 (derivation cohort) and after or during 2009 (test cohort). These time periods were chosen because both IMPACT and IHTSA were developed on patients between 1997–2008 and we wanted disjoint sets (derivation and test) to evaluate the prediction performance. The number of variables extracted from the database was 56 in total, where IHTSA uses 43 of them and IMPACT 18. The primary endpoint was one-year mortality and the second endpoint was all-cause cumulative mortality during the study period.

### Storing the Data

We converted the complete UNOS database containing heart transplants until 2011, except a few variables, into a Resource Description Framework (RDF) database following the procedure outlined in a previously published report^15^. This enabled us to use the SPARQL language to query the data and easily retrieve the variables used by both the IMPACT and IHTSA model to predict the mortality of the transplants^16^.

### Statistical Analysis

We performed the statistical analyses using the Stata MP statistical package version 13 (2013) (StataCorp LP, College Station, TX), and with RStudio Desktop 0.99.441 (RStudio, Boston, MA) using R version 3.3.1. Data are presented as means with standard deviation (SD), and frequency as appropriate. The Anderson-Darling test was used to assess the normality of the variables^17^. We used the t-test and chi-squared test for continuous, respectively categorical values, to test if the data was significantly different from each other. As with all patient registries, the dataset contains missing values. We applied a probability imputation technique by creating a list for each variable in the data set, containing the non-missing values for that variable, and then we imputed each missing value with a value from the list, chosen from a uniform distribution^18^. In consequence, the distribution of the imputed values should follow that of the non-missing ones.

The discriminatory power for one-year mortality was assessed by calculating the AUROC^19^. We compared the statistical significance of the difference between the AUROC of the two models using the non-parametric DeLong’s test^20^. To evaluate the discrimination for long-term survival of the patients, we utilised the Harrell’s concordance index (C-index)^21^. We used a z-score test to compare the C-indexes^22^. The AUROC and C-index values are both presented with 95% confidence limits. The predictive accuracy of the models was assessed by comparing the observed and expected mortality for equal-sized quantiles of risk by using the Hosmer–Lemeshow goodness-of-fit test^23^.

### The IMPACT model

IMPACT was created with a data set of heart transplant patients between 1997 to 2008 that were collected from the UNOS database. IMPACT only utilises recipient variables. Creatinine clearance was not directly available from the data set and had to be calculated using the Cockcroft-Gault equation^24^. By apportioning points according to the relative importance of the variables for the one-year mortality, a risk index was created. The minimum number of scoring points a patient can have is 0 and the maximum is 50. The points are after that converted to a predicted probability of one-year mortality by a formula derived from logistic regression^5^.

### The IHTSA model

The data set used in developing IHTSA was extracted from the ISHLT containing HT patients who were transplanted between 1994 and 2010. IHTSA utilises both recipient and donor variables. The survival model consists of a flexible nonlinear generalisation of the standard Cox proportional hazard model. Instead of using a single prediction model, this model integrates ensembles of artificial neural networks (ANNs). In addition, its prediction capability is not limited to one year^6^.

However, the variables hypertension and antiarrhythmic drugs are not recorded in the UNOS database from 2007 and onward. To handle this problem, we first imputed them with random values taken from the earlier time era. Secondly, we excluded these two variables, and retrained (calibrated) the neural network, utilizing a 5-fold cross validation of the patients between 1997 and 2008 in UNOS. The same training procedure was used as described in the original IHTSA article, but we did not carry out any new variable selection^6^. We called this model the recalibrated IHTSA model.

### Web-Based IHTSA Calculator

The IHTSA model is available via a web application (ihtsa.cs.lth.se), where a user can either input a single patient’s data or submit a file of multiple patients in a batch calculator. To compute the results, the user then selects one of the two prediction models developed either on UNOS or IHSLT data, corresponding to American or international patients respectively. The submitted file should consist of comma-separated values (CSV) reflecting the patient data in a table format. The batch calculator uses this data to predict one-, five-, and ten-year survival respectively and median survival time. Once processed, the result consisting of relevant survival and mortality numbers is either emailed back to the user in a CSV format, in the case of the batch calculator, or presented directly in the web interface.

The applications were implemented as a Java program, for the graphical user interface part and a Matlab (version 2010A and 2015b) application for running the survival models.

### Data availability

The data that support the findings of this study are available from UNOS but restrictions apply to the availability of these data, which were used under license for the current study, and so are not publicly available.
